# The Right Ventricle: From Bench to Bedside

**DOI:** 10.1155/2018/2868437

**Published:** 2018-05-14

**Authors:** Ruxandra Jurcut, Kristina Haugaa, Andre La Gerche

**Affiliations:** ^1^Institute of Emergency for Cardiovascular Diseases “Prof. Dr. C.C. Iliescu”, University of Medicine and Pharmacy “Carol Davila”, Bucharest, Romania; ^2^Oslo University Hospital, Center for Cardiological Innovation, Department of Cardiology, Unit for Cardiac Genetic Diseases, Oslo, Norway; ^3^University of Oslo, Oslo, Norway; ^4^Clinical Research Department, Baker Heart and Diabetes Institute, Melbourne, VIC, Australia

## 1. Introduction

The right ventricle (RV) remains the cardiac chamber for which scientific data regarding structure, function, adaptation to load, or arrhythmogenic potential is still behind what we know for the left ventricle, despite more recent efforts in this field. RV function is critical in numerous pathologies, related to pressure overload (like pulmonary hypertension but also arterial hypertension), volume overload (left-to-right shunts, tricuspid regurgitation), and myocardial diseases (which can be global, left ventricular, or right ventricular, more specific cardiomyopathies) as well as right ventricular ischemia or infarction. Moreover, the adaptation of the RV to more extreme physiologic situations (e.g., hypoxia at high altitude, high-level exercise) opens windows for understanding its physiology.

Despite data on the prognostic value of RV function, there is still debate on the best parameters that describe it and their clinical relevance. Important developments in right ventricular imaging have occurred during the last years, from myocardial deformation imaging to 3D-echocardiography and from cardiac MRI to right ventriculoarterial coupling studies, which have all contributed to a better understanding of right ventricular pathophysiology.

## 2. Anatomy and Physiology of the Right Ventricle

The RV is the most anterior cardiac chamber and is situated immediately behind the sternum. With a triangular shape, it has three components: inlet (sinus) portion, apical trabecular section, and outlet (conus) section. The muscular wall of the RV is normally very thin (3–5 mm), adequately serving the ejection of blood in a low impedance pulmonary circulation [1]. The RV free wall has subepicardial myofibers with transverse orientation and longitudinally arranged apex to base subendocardial myofibers [2, 3]. The middle layer of circumferential fibers seen in the left ventricle (LV) is absent in RV [4].

The shape, architecture, and structure of the RV facilitate the understanding of its physiology. Owing to the curvature of the interventricular septum (IVS) in the normal heart, the RV is described as wrapping around the LV. This particular shape, as well as the connection of the two ventricles through the IVS, constitute the interventricular dependence. In consequence, in cross section, the RV has a crescent shape which is formed because of its lower pressures, thinner walls, and greater compliance compared to the LV [5].

RV contraction has a longitudinal “peristaltic” pattern, with a 30–40 ms delay from the onset of contraction of the RV free wall from apex to the outflow tract [6], facilitating the ejection of blood to the outflow tract in the crescent shaped cavity. Under normal loading conditions, there are little short axis thickening, rotation, and twisting [7, 8].

Several studies have tried to understand the mechanisms of adaptation and maladaptation of the RV to volume and pressure overload, respectively. A few years ago, we demonstrated that at similar levels of pressure overload the RV is less dilated and performs better in patients with pulmonary stenosis as compared with those with pulmonary arterial hypertension (PAH) [9], which was in line with experimental observations. These data suggested that beyond pressure overload effects on the sarcomeric function other pathogenic factors should be taken into account. In the present issue, S. Guimaron et al. discuss the current knowledge and recent advances of RV molecular biology and metabolism from congenital heart disease to chronic PAH, with a common pathway during RV failure of metabolic glycolytic shift and altered angiogenesis.

Moreover, acute RV failure is increasingly seen in the intensive care unit and can cause or aggravate many common critical diseases. It can be due to either acute pressure or volume overload or other aggravating factors leading to a reduction of myocardial contractility owing to ischemia, cardiomyopathy, or arrhythmia [10]. J. C. Grignola and E. Domingo discuss in their paper from the present issue the mechanisms and management of acute RV dysfunction in the intensive care unit.

## 3. Pulmonary Hypertension and RV Changes

It has been demonstrated that, beyond etiology, a key element for establishing prognosis in patients with PAH is RV function [11]. The RV is especially challenged when it has to adapt to markedly (up to four- to fivefold) increased chronic afterload. According to the law of Laplace, myocardial hypertrophy allows normal wall stress, while initially preserving RV function. Over time, however, this adaptive mechanism is overrun, and contractile dysfunction and RV dilation occur, with subsequent increase in wall stress which stimulates further hypertrophy, leading to a vicious circle of declining RV performance, with ensuing RV failure and eventually death [7]. The evolution of RV failure in this setting is highly variable. Especially in the setting of congenital heart disease, as in Eisenmenger syndrome or pulmonary stenosis, RV performance may only decline slowly, showing that increased afterload is not the only determinant of RV failure [12, 13].

While pulmonary vasodilators appear to have impacted on the natural history of PAH, there have been few investigations assessing the impact of these drugs on RV remodeling. In the study of N. Rai et al. from the present issue, both sildenafil and riociguat prevented the deterioration of RV function, as determined by a decrease in RV dilation and restoration of the RV ejection fraction, while riociguat also prevented RV fibrosis induced by pulmonary artery banding (a model of fixed RV pressure overload). These experimental data need further investigation in the clinical setting.

## 4. Right Ventricular Dysfunction Secondary to Left Heart Disease

It is well known that the most frequent cause of RV failure in clinical practice is related to pulmonary hypertension due to left heart disease (e.g., systolic and diastolic LV dysfunction or left side valvular diseases) [14]. The prognostic importance of RV failure in this setting has been well demonstrated [15, 16], and data has emerged on the prognostic significance of exercise induced RV dysfunction in valvular heart disease [17]. Moreover, the association of RV dysfunction to left valvulopathies, even if not included in surgical risk scores, is often perceived as a limitation for surgery in these patients [18]. New less aggressive techniques for valvular repair, as the Mitral Clip, can surpass this barrier, and M. Hünlich et al. showed in their paper that Mitral Clip implantation improved pulmonary artery pressure, tricuspid regurgitation, and TAPSE after 12 months, while there was also a decrease in the RVOT diameter.

## 5. Right Ventricular Myocardial Changes in Specific Diseases

Various systemic or cardiac diseases can directly affect the RV. This has been described for genetic and nonhereditary cardiomyopathies. For example, diseases like hypertrophic cardiomyopathy, amyloidosis, Fabry's cardiomyopathy, and dilated cardiomyopathy can have biventricular involvement which occurs most often late during the disease evolution.

Not only cardiomyopathies but also immune and inflammatory diseases with cardiac tropism can affect the RV as well. Chagas disease is a tropical disease caused by* T. cruzi* protozoan infection, which can be at the root of more than 10% of heart failure cases in endemic regions (like Brazil). As it is often associated with systemic congestion, studies of RV involvement were started years ago, and the review of M. M. D. Romano et al. in this issue has discussed the role of imaging in the diagnosis of RV involvement in Chagas cardiomyopathy. While no specific cardiovascular imaging tools appear to assess myocardial involvement in this infectious disease, there is a place for speckle tracking imaging and cardiac MRI to identify early functional changes during the course of disease.

Systemic sclerosis (SSc) is a disease which can involve the RV in various ways. Being the leading cause of pulmonary arterial hypertension (PAH) among connective tissue diseases, it has been reported to result in increased pressure afterload of the RV in up to 12% of cases, which can subsequently result in right ventricular failure. Moreover, a direct effect of scleroderma on the myocardium consists of increased fibrosis and inflammatory lesions, and autopsy studies identified significant cardiac fibrotic changes in 70–80% of the examined patients [19]. Moreover, Hachulla et al. found that decreased left ventricular (LV) ejection fraction could be demonstrated on cardiac MRI in almost one-quarter of SSc patients. Mid-myocardial LV delayed contrast enhancement in a noncoronary distribution was also observed suggesting fibrosis mediated by an inflammatory process [20]. The paper of R. Cucuruzac et al. in the present issue provides an in-depth discussion of the current knowledge on RV remodeling and function in scleroderma patients.

## 6. Future Directions

More studies investigating the normal right ventricle structure and function and especially its adaptation to physiological states (e.g., exercise, pregnancy) and disease are warranted. While its relation to prognosis in PAH was demonstrated, quantitative parameters of RV dysfunction are not yet considered a part of risk stratification for these patients, proving insufficient knowledge of the best parameter to follow.

*Ruxandra Jurcut* *Kristina Haugaa* *Andre La Gerche*
